# Electronic control of H^+^ current in a bioprotonic device with carbon nanotube porins

**DOI:** 10.1371/journal.pone.0212197

**Published:** 2019-02-22

**Authors:** Zahra Hemmatian, Ramya H. Tunuguntla, Aleksandr Noy, Marco Rolandi

**Affiliations:** 1 Department of Electrical and Computer Engineering, University of California Santa Cruz, Santa Cruz, CA, United States of America; 2 Physics and Life Sciences Directorate, Lawrence Livermore National Laboratory, Livermore, CA, United States of America; 3 School of Natural Sciences, University of California Merced, Merced, CA, United States of America; Northeastern University, UNITED STATES

## Abstract

Hybrid biotic abiotic devices can be used to interface electronics with biological systems for novel therapies or to increase device functionality beyond silicon. Many strategies exist to merge the electronic and biological worlds, one dominated by electrons and holes as charge carriers, the other by ions. In the biological world, lipid bilayers and ion channels are essential to compartmentalize the cell machinery and regulate ionic fluxes across the cell membrane. Here, we demonstrate a bioelectronic device in which a lipid bilayer supported on H^+^-conducting Pd/PdH_x_ contacts contains carbon nanotubes porin (CNTP) channels. This bioelectronic device uses CNTPs to control of H^+^ flow across the lipid bilayer with a voltage applied to the Pd/PdH_x_ contacts. Potential applications of these devices include local pH sensing and control.

## Introduction

Bioelectronic devices that interface with biological systems have many potential applications including new therapies and computational systems with functionalities beyond silicon [1, 2]. Examples include electroceuticals [3], wearables [4], electronic plants [5], and edible electronics [6]. In biological systems, membrane proteins and ion channels contribute to most of the communication between cells and their environments. Ion channels either passively allow or actively control the flow of ions, typically Na^+^, K^+^, Cl^-^, and Ca^2+^, and small molecules across the cell membrane [7]. Although protons are not directly involved in neuronal action potential generation and propagation, proton (H^+^) currents and concentration,[H^+^], gradients play essential physiological roles in a number of other processes [8]. The most striking example is oxidative phosphorylation in mitochondria in which proton gradients serve as a means to translate the energy from oxidation of glucose during the Kreb’s cycle into ATP, the biological energy currency [9, 10]. Other examples include the light-activated H^+^ pumping by archaeal bacteriorhodopsins [11], the activation of bioluminescence from H^+^ in dinoflagellates [12], the bacterial flagellar motor activation [13], and the activity of the antibiotic Gramicidin [14]. Recently, carbon nanotube porins (CNTPs) have emerged as artificial channels that can conduct protons across lipid bilayers [15]. In particular, narrow sub-1-nm diameter CNTPs that force water into a single-file wire conformation show very high H^+^ conductivity exceeding that of Gramicidin channel and even exceeding the intrinsic conductance of Nafion [16].

Man-made electronic platforms, which use electronic currents to carry charge have an intrinsic difficulty connecting to the biological systems that rely mostly on ionic currents [17]. To this end, many efforts in bioelectronics focus on strategies to interface ionic and electronic signaling. For example, researchers showed carbon nanotube, silcon nanowire, and organic field effect transistor devices that integrated gramicidin and rhodospins as gating elements [18–20]. Organic bioelectronics[21–22] with mixed ionic [23–25] and electronic conductivity enables devices that can both record and stimulate physiological function, and can be assembled into logic circuits [26]. We have recently demonstrated bioprotonic devices that control the flow of H^+^ in field effect transistors (H^+^-FETs) [27–30] and memories [31], and integrated these devices with enzymes to create logic gates [28]. H^+^ conducting transistors with squid reflectin proteins have also been described [32–34]. All of these H^+^ conducting devices incorporated Pd/PdH_x_ contacts, which translated an H^+^ current into an electrical response [30,35].

**Fig 1:**
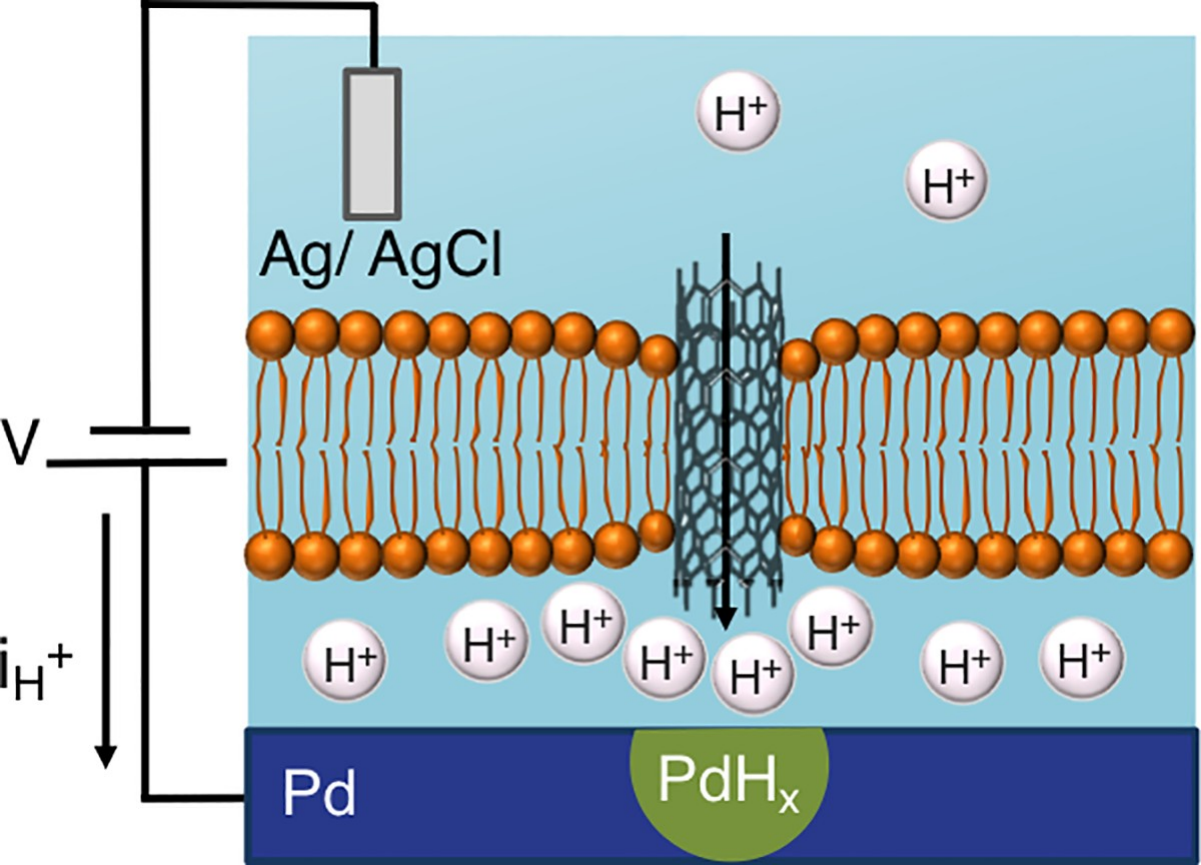
A bioprotonic device with integrated carbon nanotube porins (CNTPs) supports proton current across the SLB through the CNTPs when a negative voltage (*-V*) is applied on the Pd contact. When H^+^ reach the surface of the Pd contact, they are reduced to H by an incoming electron and diffuse into the Pd to form palladium hydride (PdH_x_). The current density at the contact (–*i*_H+_), measures the rate of H^+^ flux along the CNTPs.

We have previously demonstrated the integration of gramicidin, alamethicin, and deltarhodpsin with Pd/PdH_x_ contacts and created devices that control H^+^ currents and modulate pH gradients across phospholipid membranes [36, 37]. Here, we expand these types of devices to a fully synthetic platform by using CNTPs as H^+^ channels mimics (Fig 1). These devices comprise a supported lipid bilayer (SLB) that mimics the function of a cell membrane at the Pd/solution interface and acts as a self-sealing support for the insertion of the CNTP channels.

## Materials and methods

### Devices

We fabricated bioprotonic devices as previously described [36,37]. In brief, we defined a Pd contact area ranging from 10 μm^2^ (2 μm × 5 μm) to 200 μm^2^ (2 μm × 100 μm) by evaporating 100nm Pd with a 5nm Cr as an adhesive layer onto a Si wafer with a 100 nm SiO_2_ insulating layer. A photolithographically patterned SU-8 photoresist defined a microfluidic channel over the device. A polydimethylsiloxane (PDMS) well was placed on top of the microfluidic channel to confine the solution and to provide enough room to insert the Ag/ AgCl electrode that acts as a counter and a reference electrodes. The channel was filled with a buffered solution of different strength and composition as described in the Results section.

### Liposome and CNTPs Liposome

Liposomes were prepared using a 1,2-Dioleoyl-sn-glycero-3-phosphocholine (DOPC, Sigma Aldrich Lipids). We followed a hydration- dehydration protocol to form liposomes. The lipids were dissolved in chloroform into glass vials and became dehydrated by evaporation of the solvent under a stream of argon gas. To dry the lipids further we stored them in a vacuum desiccator chamber overnight. Liposomes were prepared by hydrating the lipids with a buffer solution containing 10 mM HEPES, 150 mM NaCl, 30 mM KCl pH = 7.0 to the dried lipid film to obtain a final lipid concentration of 2 mg ml^-1^. This solution was hydrated at room temperature and bath-sonicated for 30 min. Liposomes were then extruded 20 times through a 200 nm (LUVs) pore-sized polycarbonate membrane using a mini-extruder (Avanti Polar Lipids). We used Dynamic Light Scattering (DLS) to characterize the size of liposomes.

To incorporate 1.5 or 0.8 nm diameter CNTPs into the liposomes, we first dissolved appropriate amount of DOPC–CNTP complex into 2 ml solution. We then kept the mixture in a vacuum desiccator overnight to remove the solvent. The dried DOPC–CNTP complex was hydrated with 10 mM HEPES, 150 mM NaCl and 30 mM KCl at pH 7.0 and bath-sonicated until completely solubilized. We prepared a dried lipid film in a separate glass vial, hydrated it using the solubilized DOPC-CNT complex, and bath-sonicated the solution to obtain a final lipid concentration of 2 mg ml^-1^. The mixture was extruded through a 200 nm polycarbonate filter using a mini-extruder (Avanti Polar Lipids). The vesicle size was characterized using a DLS instrument (Malvern).

### Supported Lipid Bilayers (SLB)

We deposited DOPC or DOPC-CNTP liposomes solution onto the Pd contact and formed an SLB over it using vesicle fusion. This SLB mimics a cell membrane, electrically insulates the Pd contact (ρ ~ 3 G Ω cm^-1^) and divides the solution into two volumes. We refer to the larger volume containing the Ag/AgCl electrode as the bulk solution (B). We refer to the small volume between the SLB and the Pd contact as the isolation layer (IL).

### Atomic Force Microscopy (AFM)

We used an MFP-3D Origin^TM^ (Oxford Instruments- Asylum Research) operating in air at room temperature, in conductive atomic force microscope (c-AFM) mode, which measures the current through the tip as a function of applied voltage, V. To preserve the integrity of the SLB coating we maintained high relative humidity of 75%. At this high relative humidity and without drying the sample, we postulate that a thin layer of water is still present on the SLP and keeps it stable. For sample preparation, excess buffer solution was gently removed from the surface of the Pd contact. The sample then was placed into a conductive sample holder to complete the electric circuit. The tip position was controlled using a custom-made LabView program that converts graphic information to voltage commands, which are sent to the AFM scanner. Current measurements were performed using a dual-phase amplifier with the amplitude of -250 mV and frequency of 0.99 Hz.

## Results and discussion

In our bioprotonic devices, the Pd contact acts as a working electrode and the Ag/AgCl acts as a counter and reference electrode. For a bare Pd contact, when we apply a DC voltage difference between the Pd contact and the Ag/AgCl electrodes (V), we measure the resulting proton current (i_H+_). For -V applied to the Pd contact, H^+^ flow from the solution onto the Pd surface, where they are reduced to H by an e^-^. These H^+^ leave behind a OH^-^ ions that make the pH of the solution increase for large enough currents as we have previously demonstrated. H then absorb into the Pd to form PdHx. Conversely, for +V, the H in PdH oxidizes into H^+^ at the Pd/solution interface and are released into solution.

**Fig 2:**
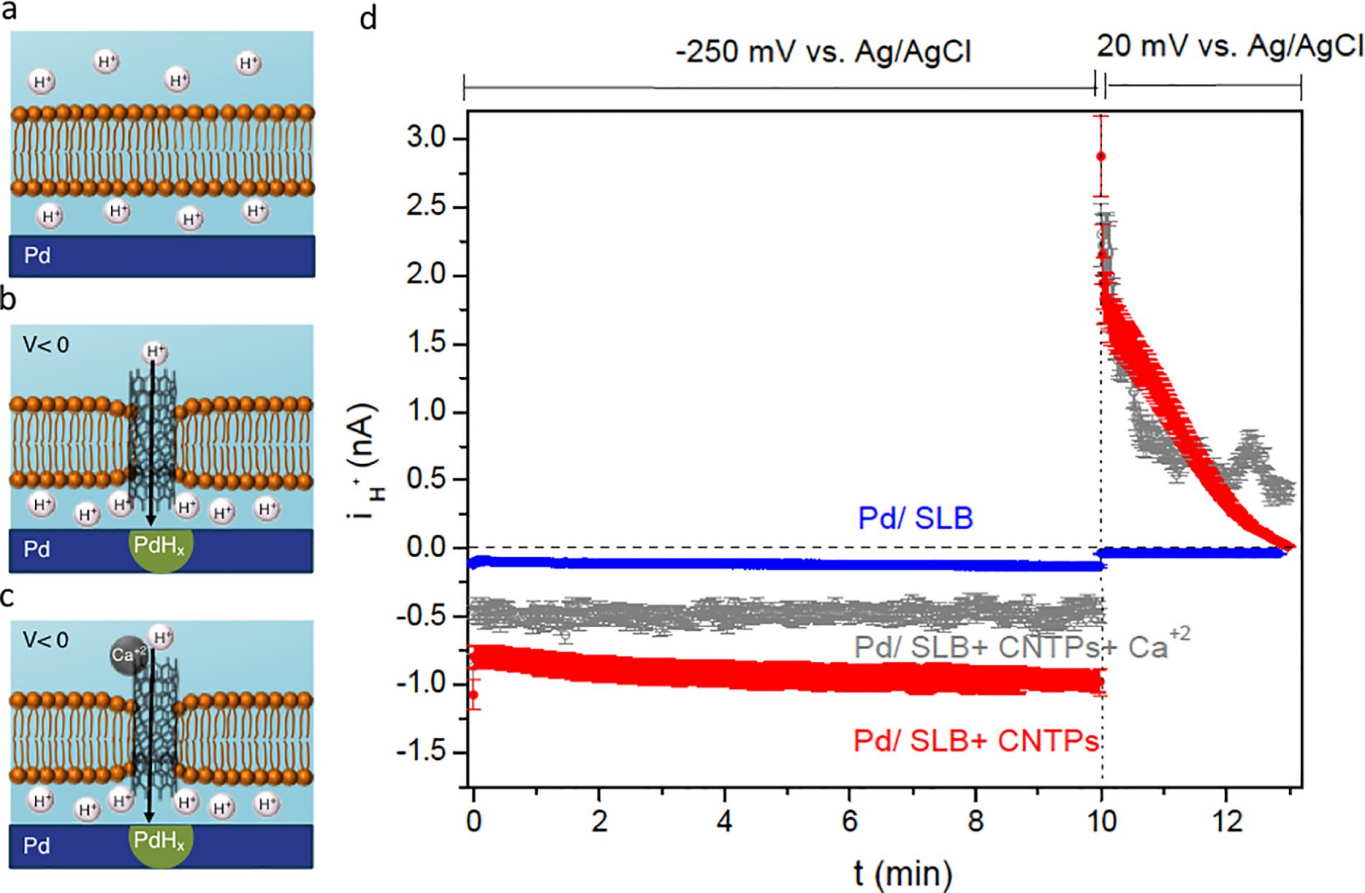
(**a**) Pd contact with SLB. The SLB blocks H^+^ from transferring from the solution to the Pd contact even with V = -250 mV (vs. Ag/AgCl). (**b**) Pd contact with SLB incorporating 0.8 nm diameter CNTPs is semipermeable to H^+^, with CNTPs facilitating the rapid flow of H^+^ to the Pd/solution interface. (**c**) Upon addition of Ca^+2^ to the bulk solution, H^+^ current through CNTPs becomes partially blocked. (d) *i*_H+_ vs. time plots recorded at *V* = −250 mV and *V* = 20 mV. Blue trace: SLB, red trace: SLB with CNTPs, gray trace SLB with CNTPs in presence of Ca^+2^ ions in the bulk solution. (The data are collected from 3 different devices with different dimensions: Pd / SLB: 3 different devices of 2 × 50 μm, Pd/SLB+CNTPs: 3 different devices of 2 × 50 μm, Pd/SLB+CNTPs+Ca^+2^: 3 different devices of 2 × 50 μm. The error bars are the root mean square of the displacement of the data from the average value).

After we formed a supported lipid bilayer (SLB) membrane with integrated CNTPs on the Pd contacts, applied voltage to the contact and measured i_H+_. As control, with the Pd contact protected by the SLB in the absence of CNTPs, applied voltage of *V* = -250 mV resulted in a negligible *i*_H+_ = -0.06 ± 0.01 nA (Fig 2A and 2D). This amount of *i*_H+_ indicates that the SLB creates an effective barrier that minimizes transport of H^+^ to the Pd contact surface. To confirm this result, we set *V* = 20 mV after applying *V* = -250 mV for 10 minutes. If any H^+^ crossed the SLB with *V* = -250 mV, they will reduce onto the surface and diffuse into the Pd to form PdH_x_ [28]. This PdH_x_ has a higher protochemical potential (μ_H+_) than the *pH* = 7.0 solution at *V* = 20 mV. As a result of this higher μ_H+_ for the PdH_x_ contact, H would oxidize at the PdH_x_ contact solution interface and a proton current would flow from the PdH_x_ contact into the solution at the IL. This current would be measured as *i*_H+_[28] with a positive value. The blue trace in Fig 2D shows that this is clearly not the case.

In contrast, when we inserted CNTPs in the SLB, i_H+_ was much larger with *V* = -250 mV and *i*_H+_ continually increasing with *i*_H+_ = -1.12 ± 0.05 nA at *t* = 10 min. (Fig 2B and 2D). This large i_H+_ confirms our conjecture that the CNTPs inserted in the SLB support the H^+^ current. To verify this conjecture, we added 1 mM Ca^+2^ ions, which were previously demonstrated to block H^+^ from entering CNTPs [16] and carrying current across the SLB (Fig 2C) [19]. As expected, *i*_H+_ values recorded at *V* = -250 mV is i _H+_ = – 0.47 ± 0.03 nA (Fig 2D, grey trace).

**Fig 3:**
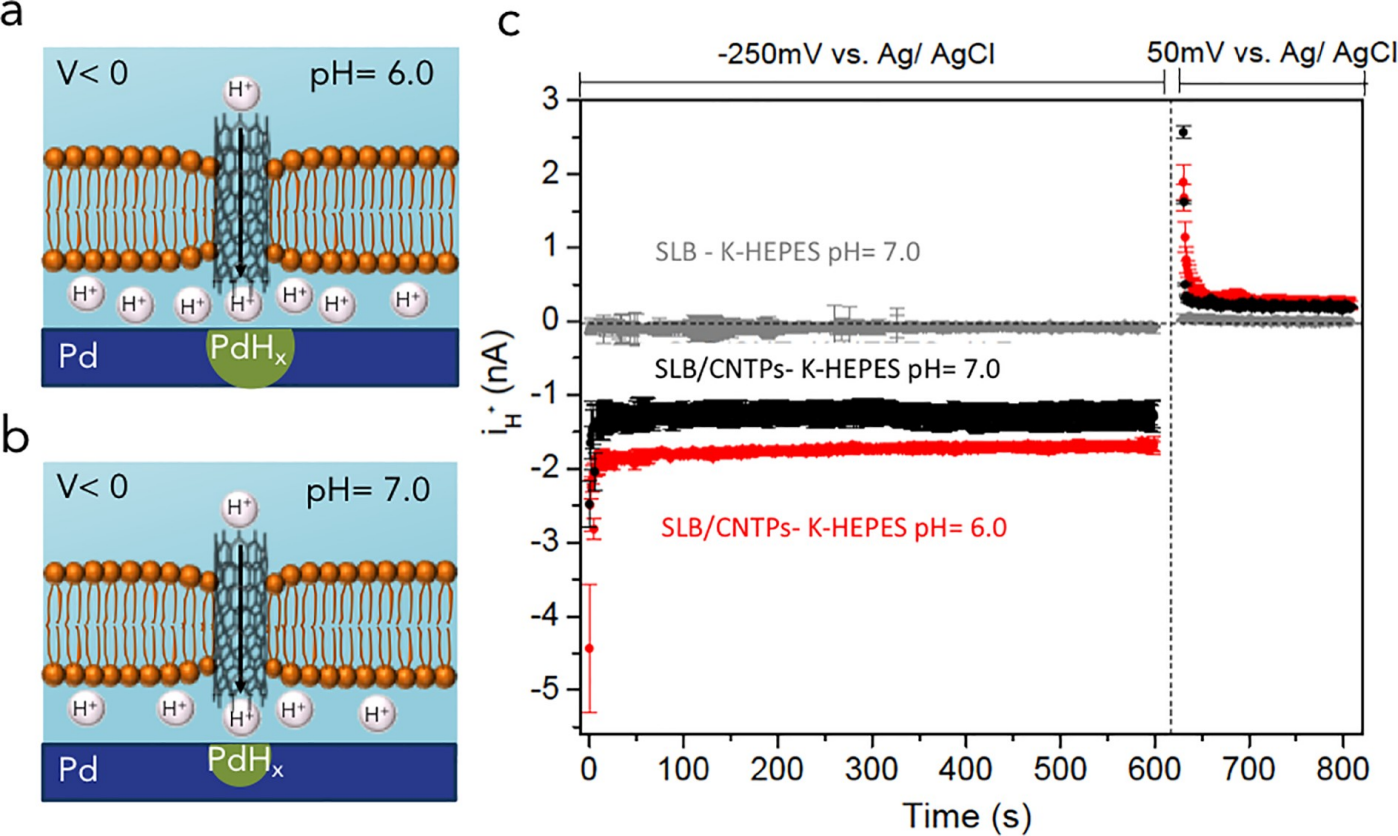
(**a**) Pd contact with SLB incorporating 0.8 nm diameter CNTPs with K-HEPES buffer at pH = 6.0, is semipermeable to H^+^, with CNTPs facilitating the rapid flow of H^+^ to the Pd/solution interface. (**b**) Pd contact with SLB incorporating Narrow CNTPs with K-HEPES buffer pH = 7.0, is still semipermeable to H^+^ but facilitating lower flow of H^+^ to the Pd/solution interface. (**c**) *i*_H+_ versus time plot for *V* = −250 mV and *V* = 50 mV. Gray trace SLB, red trace SLB+ CNTps (K-HEPES, pH = 6.0), black trace SLB+ CNTPs (K-HEPES, pH = 7.0). The *i*_H+_ for measurements K-HEPES pH = 6.0 is higher than K-HEPES pH = 7. We can hypothesize that at pH = 6.0 we have a driving force due to the lower pH across the membrane in addition to the applied voltage that expedite the flow of H^+^ while at pH = 7.0 we have only the applied voltage as a driving force to transport the H^+^ across. We did not observe any significant different between the *i*_H+_ at pH = 8.0 as compare to pH = 7.0 which might be due the buffer capacity of HEPES at different pH condition (Fig A in S1 File). (The data are collected from 3 different devices with different dimensions: SLB- K-HEPES pH = 7.0 : 3 different devices of 2 × 50 μm, Pd/SLB+CNTPs+Ca^+2^: 3 different devices of 2 × 50 μm. The error bars are the root mean square of the displacement of the data from the average value).

To confirm that H^+^ are indeed the carriers for the observed for i_H+_, we performed the experiments with different buffers that maintain pH = 7.0 or pH = 6.0 (Fig 3A, Fig 3B). Using the same V sequence that we used in Fig 2, we consistently measured i_H+_ to be higher at pH = 6.0 (i_H+_ = -2.08 ± 0.02 nA nA) (Fig 3C, red trace) than at pH = 7.0 (i_H+_ = -1.58 ± 0.04 nA nA) (Fig 3C, black trace). This is expected because lower pH value corresponds to higher H^+^ concentration for H^+^ current to flow. To exclude the possibility of the current being caused by the ionic flux through the CNTPs, we also performed experiments with buffer with and without K^+^ ions. At the same pH, we observed very little difference between i_H+_ whether K^+^ ions were present, i_H+_ = 1.10 ± 0.05 nA or not i_H+_ = 1.16 ± 0.04 nA (Fig A in S1 File)., indicating that K^+^ ions do not significantly contribute to i_H+_. Note that if K^+^ ion current through CNTPs is, then even larger ions would provide an even smaller contribution to the device current.

**Fig 4:**
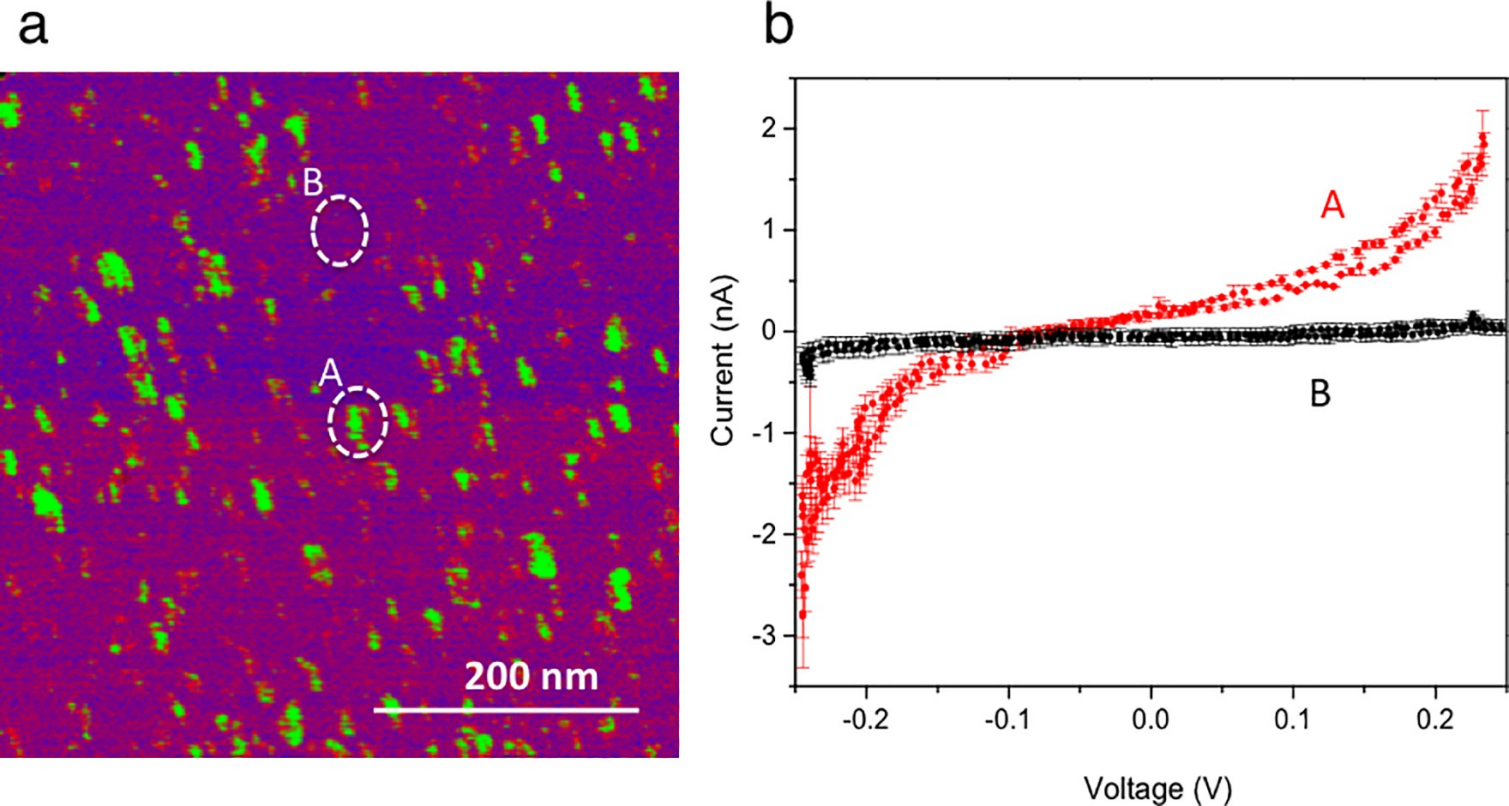
Conductive AFM of SLB with CNTPs channels. (a) The current map for the Pd contact with SLB incorporating CNTPs. The hot spot (green spot) correspond to higher current (red trace) that represent CNT and the background (purple area) correspond to negligible amount of current (black trace) which represent SLB membrane. (b) In the IV curve the red trace collected from the green spot and the back trace collected from the purple area. The green spot has i ~ 1.78 nA ± 0.09 nA and purple area has i ~ 5.86 pA ± 0.98 pA. This current most likely represents the electron conductivity of CNT. (The data are collected from 3 different areas of the AFM image for both green spot and purple area. The error bars are the root mean square of the displacement of the data from the average value).

To confirm the presence of CNTPs in the lipid bilayer, we performed conductive AFM imaging to generate a current map (Fig 4A). In this map, green spots correspond to areas where the current measured by the conductive AFM tip is larger than the background, shown in purple. When the AFM tip is placed on top of a CNT indicated by a hot spot shown in green, the measured current is as high as i = 1.78 nA ± 0.09 nA with V = 250 mV applied between the tip and the Pd contact (Fig 4B, red trace (A)). When the tip is placed on the top of the lipid bilayer in a region associated with purple on the map, minimal current of i = 5.86 pA ± 0.98 pA is measured (Fig 4B, black trace (B)) confirming that the supported lipid bilayer is a good insulator for the Pd contact. The current measured in Fig 4B is likely electronic current due to the direct contact of the CNTPs with the tip, which by pressing on the CNTP also creates a contact with the CNTP and the Pd/PdH_x_ contact. There is likely a component of H^+^ current but we estimate that this component is negligible. From these images, we estimate 1,000 CNTPs per contact, thus the H^+^ current per CNTP as measured with the bioprotonic device is ~ 1 pA per CNTPs, which is much smaller than the one recorded in Fig 4B. Nonetheless, the proton current measured with the CNTPs integrated in the SLB is comparable with what have measured for gramicidin [36].

## Conclusions

In conclusion, we have demonstrated that CNTPs can act as gramicidin mimics when integrated in a bioprotonic device formed by Pd contacts with a single lipid bilayer membrane. Using buffers with different H^+^ concentration and K^+^ concentration we have confirmed that CNTPs in our devices predominantly conduct a current of H^+^. Given the ability of CNTPs to penetrate cells, and their high intrinsic proton conductance CNTPs may be used to connect bioprotonic devices directly with cells to modulate intracellular pH or to create localized pH gradients in solution.

## Supporting information

S1 File Electronic control of H+ current in a bioprotonic device with carbon nanotubes. **Fig A.** (a) Pd contact with SLB incorporating Narrow CNT (0.8nm) with HEPES buffer at pH = 7.0, is semipermeable to H+, with CNT facilitating the rapid flow of H+ to the Pd/solution interface. (b) Pd contact with SLB incorporating Narrow CNT with K-HEPES buffer pH = 7.0, is still semipermeable to H+ and facilitating flow of H+ to the Pd/solution interface. (c) iH+ versus time plot for V = −250 mV and V = 50 mV. Gray trace SLB, red trace SLB+ CNT (K-HEPES), black trace SLB+ CNT (HEPES). The change in iH+ for measurements with potassium (K-HEPES) and without potassium (HEPES) is negligible. (The data are collected from 3 different devices with different dimensions: SLB- K- KEPES pH = 7.0: 3 different devices of 2 × 50 μm, SLB/ CNTPs- K- KEPES pH = 6.0: 3 different devices of 2 × 50 μm, SLB/ CNTPs- K- KEPES pH = 7.0: 3 different devices of 2 × 50 μm. The error bars are the root mean square of the displacement of the data from the average value).
(DOCX)

## References

[1] Malliaras G, Abidian MR. Organic Bioelectronic Materials and Devices. Advanced Materials. 2015;27(46):7492–. 10.1002/adma.201504783 26636956 PMC4682871

[2] Noy A. Mimicking Biology with Nanomaterials: Carbon Nanotube Porins in Lipid Membranes. Biophysical Journal. 2015;108(2):443a.

[3] Jonsson A, Song Z, Nilsson D, Meyerson BA, Simon DT, Linderoth B, et al Therapy using implanted organic bioelectronics. Science Advances. 2015;1(4):e1500039 10.1126/sciadv.1500039 26601181 PMC4640645

[4] Pang C, Koo JH, Nguyen A, Caves JM, Kim MG, Chortos A, et al Highly Skin‐Conformal Microhairy Sensor for Pulse Signal Amplification. Advanced Materials. 2015;27(4):634–40. 10.1002/adma.201403807 25358966

[5] Stavrinidou E, Gabrielsson R, Gomez E, Crispin X, Nilsson O, Simon DT, et al Electronic plants. Science Advances. 2015;1(10):e1501136 10.1126/sciadv.1501136 26702448 PMC4681328

[6] Kim YJ, Chun S-E, Whitacre J, Bettinger CJ. Self-deployable current sources fabricated from edible materials. Journal of Materials Chemistry B. 2013;1(31):3781–8. 10.1039/c3tb20183j 32261130

[7] Shen Y-x, Saboe PO, Sines IT, Erbakan M, Kumar M. Biomimetic membranes: A review. J Membr Sci. 2014;454:359–81.

[8] DeCoursey TE. The voltage-gated proton channel: a riddle, wrapped in a mystery, inside an enigma. Biochemistry. 2015. 10.1021/acs.biochem.5b00353 PMC4736506 25964989

[9] Mitchell P. Proton-translocation phosphorylation in mitochondria, chloroplasts and bacteria: natural fuel cells and solar cells. Federation proceedings. 1967;26(5):1370–9. 4228052

[10] Morowitz HJ. Proton semiconductors and energy transduction in biological systems. American Journal of Physiology-Regulatory, Integrative and Comparative Physiology. 1978;235(3):R99–R114. 10.1152/ajpregu.1978.235.3.R99 696856

[11] Lanyi JK. Bacteriorhodopsin. Annu Rev Physiol. 2004;66:665–88. 10.1146/annurev.physiol.66.032102.150049 14977418

[12] Smith SM, Morgan D, Musset B, Cherny VV, Place AR, Hastings JW, et al Voltage-gated proton channel in a dinoflagellate. Proc Natl Acad Sci U S A. 2011;108(44):18162–7. 10.1073/pnas.1115405108 22006335 PMC3207696

[13] Walz D, Caplan SR. Bacterial flagellar motor and H+/ATP synthase: two proton-driven rotary molecular devices with different functions. Bioelectrochemistry. 2002;55(1):89–92. 11786348 10.1016/s1567-5394(01)00162-1

[14] Busath D, Szabo G. Gramicidin forms multi-state rectifying channels. 1981 6171731 10.1038/294371a0

[15] Geng J, Kim K, Zhang J, Tunuguntla R, Comolli L, Allen F, et al Stochastic transport through carbon nanotubes in lipid bilayers and live cell membranes. Nature. 2014;514:612–5. 10.1038/nature13817 25355362

[16] Tunuguntla R, Allen F, Kim K, Bellivieau A, Noy A. Ultrafast proton transport in sub-1-nm diameter carbon nanotube porins. Nat Nanotechnol. 2016; 11:639–44. 10.1038/nnano.2016.43 27043198

[17] Ajo‐Franklin CM, Noy A. Crossing Over: Nanostructures that Move Electrons and Ions across Cellular Membranes. Advanced Materials. 2015. 10.1002/adma.201500344 25914282

[18] Huang S-CJ, Artyukhin AB, Misra N, Martinez JA, Stroeve PA, Grigoropoulos CP, et al Carbon nanotube transistor controlled by a biological ion pump gate. Nano letters. 2010;10(5):1812–6. 10.1021/nl100499x 20426455

[19] Misra N, Martinez JA, Huang S-CJ, Wang Y, Stroeve P, Grigoropoulos CP, et al Bioelectronic silicon nanowire devices using functional membrane proteins. Proceedings of the National Academy of Sciences. 2009;106(33):13780–4. 10.1073/pnas.0904850106 PMC2728971 19667177

[20] Angione MD, Cotrone S, Magliulo M, Mallardi A, Altamura D, Giannini C, et al Interfacial electronic effects in functional biolayers integrated into organic field-effect transistors. Proceedings of the National Academy of Sciences of the United States of America. 2012;109(17):6429–34. 10.1073/pnas.1200549109 22493224 PMC3340085

[21] Johnson N, Kim YJ, Dingh H, LeDuc P, Bettinger C. Bio-Inspired pH Responsive Hydrogels for Programmed Activation of Electrochemical Storage Systems in Biology. Biophysical Journal. 2015;108(2):485a.

[22] Mitraka E, Kergoat L, Khan Z, Fabiano S, Douhéret O, Leclère P, et al Solution processed liquid metal-conducting polymer hybrid thin films as electrochemical pH-threshold indicators. Journal of Materials Chemistry C. 2015;3(29):7604–11.

[23] Owens RM, Malliaras GG. Organic Electronics at the Interface with Biology. Mrs Bulletin. 2010;35(6):449–56.

[24] Tybrandt K, Larsson KC, Richter-Dahlfors A, Berggren M. Ion bipolar junction transistors. Proceedings of the National Academy of Sciences. 2010;107(22):9929–32. 10.1073/pnas.0913911107 PMC2890459 20479274

[25] Williamson A, Rivnay J, Kergoat L, Jonsson A, Inal S, Uguz I, et al Epilepsy Treatment: Controlling Epileptiform Activity with Organic Electronic Ion Pumps (Adv. Mater. 20/2015). Advanced Materials. 2015;27(20):3097–. 10.1002/adma.201500482 25866154

[26] Tybrandt K, Forchheimer R, Berggren M. Logic gates based on ion transistors. Nature communications. 2012;3:871 10.1038/ncomms1869 22643898

[27] Deng Y, Josberger E, Jin J, Rousdari AF, Helms BA, Zhong C, et al H+-type and OH−-type biological protonic semiconductors and complementary devices. Scientific reports. 2013;3. 10.1038/srep02481 PMC3789148 24089083

[28] Miyake T, Josberger EE, Keene S, Deng Y, Rolandi M. An enzyme logic bioprotonic transducer. APL Materials. 2015;3(1):014906.

[29] Miyake T, Rolandi M. Grotthuss mechanisms: from proton transport in proton wires to bioprotonic devices. Journal of Physics: Condensed Matter. 2015;28(2):023001 10.1088/0953-8984/28/2/023001 26657711

[30] Zhong C, Deng Y, Roudsari AF, Kapetanovic A, Anantram M, Rolandi M. A polysaccharide bioprotonic field-effect transistor. Nature communications. 2011;2:476 10.1038/ncomms1489 21934660

[31] Josberger EE, Deng Y, Sun W, Kautz R, Rolandi M. Two‐Terminal Protonic Devices with Synaptic‐Like Short‐Term Depression and Device Memory. Advanced materials. 2014;26(29):4986–90. 10.1002/adma.201400320 24789251

[32] Ordinario DD, Phan L, Walkup IV WG, Jocson J-M, Karshalev E, Hüsken N, et al Bulk protonic conductivity in a cephalopod structural protein. Nature chemistry. 2014;6(7):596–602. 10.1038/nchem.1960 24950329

[33] Rolandi M. Bioelectronics: A positive future for squid proteins. Nature chemistry. 2014;6(7):563–4. 10.1038/nchem.1980 24950323

[34] Ordinario DD, Phan L, Jocson J-M, Nguyen T, Gorodetsky AA. Protonic transistors from thin reflectin films. APL Materials. 2015;3(1):014907.

[35] Glasser L. Proton Conduction and Injection in Solids. Chemical Reviews. 1975;75(1):21–65.

[36] Hemmatian Z, Keene S, Josberger E, Miyake T, Arboleda C, Soto-Rodriguez J, et al Electronic control of H+ current in a bioprotonic device with Gramicidin A and Alamethicin. Nature Communications. 2016;7. 10.1038/ncomms12981 PMC5059763 27713411

[37] Soto-Rodriguez J, Hemmatian Z, Josberger EE, Rolandi M, Baneyx F. A Palladium-Binding Deltarhodopsin for Light-Activated Conversion of Protonic to Electronic Currents. Advanced Materials. 2016;28(31):6581–+. 10.1002/adma.201600222 27185384

