# Supplementary material for: Electronic control of H+ current in a bioprotonic device with carbon nanotube porins
Source: PLoS One. 2019 Feb 22;14(2):e0212197. doi: 10.1371/journal.pone.0212197 (PMC6386364; doi:10.1371/journal.pone.0212197)
Supplement: S1 File — Fig A. (a) Pd contact with SLB incorporating Narrow CNT (0.8nm) with HEPES buffer at pH = 7.0, is semipermeable to H+, with CNT facilitating the rapid flow of H+ to the Pd/solution interface. (b) Pd contact with SLB incorporating Narrow CNT with K-HEPES buffer pH = 7.0, is still semipermeable to H+ and facilitating flow of H+ to the Pd/solution interface. (c) iH+ versus time plot for V = −250 mV and V = 50 mV. Gray trace SLB, red trace SLB+ CNT (K-HEPES), black trace SLB+ CNT (HEPES). The change in iH+ for measurements with potassium (K-HEPES) and without potassium (HEPES) is negligible. (The data are collected from 3 different devices with different dimensions: SLB- K- KEPES pH = 7.0: 3 different devices of 2 × 50 μm, SLB/ CNTPs- K- KEPES pH = 6.0: 3 different devices of 2 × 50 μm, SLB/ CNTPs- K- KEPES pH = 7.0: 3 different devices of 2 × 50 μm. The error bars are the root mean square of the displacement of the data from the average value). (DOCX) [file pone.0212197.s001.docx]

Electronic control of H+ current in a bioprotonic device with carbon

nanotubes (Supporting Information)

Fig. A. (a) Pd contact with SLB incorporating Narrow CNT (0.8nm) with HEPES buffer at

pH= 7.0, is semipermeable to H+, with CNT facilitating the rapid flow of H+ to the Pd/solution

interface. (b) Pd contact with SLB incorporating Narrow CNT with K-HEPES buffer pH= 7.0, is still semipermeable to H+ and facilitating flow of H+ to the Pd/solution interface. (c) iH+ versus time plot for V=−250 mV and V=50 mV. Gray trace SLB, red trace SLB+ CNT (K-HEPES), black trace SLB+ CNT (HEPES). The change in iH+ for measurements with potassium (K-HEPES) and without potassium (HEPES) is negligible. (The data are collected from 3 different devices with different dimensions: SLB- K- KEPES pH=7.0: 3 different devices of 2 × 50 μm, SLB/ CNTPs- K- KEPES pH=6.0: 3 different devices of 2 × 50 μm, SLB/ CNTPs- K- KEPES pH=7.0: 3 different devices of 2 × 50 μm. The error bars are the root mean square of the displacement of the data from the average value).”
